# Temperature Safeguards and Peri‐Procedural Strategies for Stem Cell Collection in Cold Agglutinin Disease: A Case Report and Literature Review

**DOI:** 10.1002/jca.70158

**Published:** 2026-07-23

**Authors:** S. Bellegarde, C. Scott, R. Sabol, T. Eunson, Z. J. Gahvari, C. P. Onyenekwu

**Affiliations:** ^1^ Department of Pathology and Laboratory Medicine, Beth Israel Deaconess Medical Center, Harvard Medical School Boston Massachusetts USA; ^2^ Department of Pathology and Laboratory Medicine University of Wisconsin Hospital and Clinics Madison Wisconsin USA; ^3^ Infusion Center and Apheresis University of Wisconsin Hospital and Clinics Madison Wisconsin USA; ^4^ Cellular Therapy Laboratory University of Wisconsin Hospital and Clinics Madison Wisconsin USA; ^5^ Department of Medicine University of Wisconsin‐Madison, and University of Wisconsin Hospital and Clinics Madison Wisconsin USA; ^6^ Department of Pathology and Laboratory Medicine University of Wisconsin‐Madison Madison Wisconsin USA

**Keywords:** apheresis, autologous transplant, cold agglutinin disease, cryopreservation, hematopoietic stem cell collection, red cell agglutination, thermal management

## Abstract

Cold agglutinins can result in red blood cell (RBC) agglutination and lysis at low temperatures. Temperature sensitivity during stem cell mobilization, processing, and infusion poses unique procedural challenges in patients with active cold agglutinins. A 49‐year‐old man presented at an outside hospital with severe hemolysis and was found to have an anti‐I cold agglutinin and a component of warm autoimmune hemolytic anemia, together with a positive COVID and Rhinovirus test. He was treated with RBC transfusions, steroids, and Rituximab. Bone marrow evaluation several months later at our institution demonstrated IgA‐kappa multiple myeloma and a rare, atypical B‐cell population of unclear significance. After induction therapy, autologous peripheral blood stem cell transplantation was recommended. The primary concerns were cold‐induced agglutination during apheresis collection of hematopoietic progenitor cells and during graft handling. This required coordination across hematology, apheresis, and cell therapy teams. The patient received cyclophosphamide for cytoreduction, granulocyte colony‐stimulating factor for mobilization, and sutimlimab for complement inhibition. The collection was performed using practical temperature mitigation strategies including environmental warming and insulation of extracorporeal components. The product was processed using prewarmed equipment, and cryopreservation was performed per institutional protocol. A total of 5.61 × 10^6^ CD34^+^ cells/kg were collected without circuit alarms or visible agglutination. Conditioning with high‐dose melphalan and autologous infusion was completed without complication, with successful engraftment. This case highlights the feasibility of pragmatic, multidisciplinary temperature management strategies during stem cell collection and processing in patients with cold agglutinin disease.

## 1 Introduction

Cold agglutinins disease (CAD) and cold agglutinin syndrome (CAS) are both autoimmune hemolytic anemias mediated by cold‐reactive antibodies, typically immunoglobulin M, that trigger complement‐dependent red blood cell destruction at reduced temperatures, but differ in etiology. While CAD is a primary lymphoproliferative disorder, CAS is a secondary condition associated with infections such as Mycoplasma pneumonia and other malignancies. Although the clinical features and implications for transfusion management are well documented, managing cold agglutinins (CA) is particularly challenging in the setting of extracorporeal therapies. In patients requiring peripheral blood stem cell (PBSC) collection and autologous stem cell transplantation, hypothermia during apheresis, processing, and infusion can precipitate clinically significant hemolysis. Published experience in this setting remains limited, with only a small number of reported cases, most of which involve classic IgM‐mediated CAD [1, 2, 3, 4, 5].

Beyond the extracorporeal circuit, the patient's environmental exposure, product collection bag handling, transport, and processing are vulnerable to temperature‐related factors. Previous reports have described red blood cell (RBC) clumping in PBSC bags, demonstrating that cold‐induced agglutination can occur despite stable intraprocedural apheresis parameters [6]. Experimental and case studies have also evaluated strategies for warming and processing hematopoietic progenitor cell apheresis (HPC‐A) products [7, 8].

Here, we report a case of successful PBSC collection in a patient with clinically significant cold agglutinins and IgA κ–myeloma undergoing autologous stem cell transplantation, highlighting diagnostic considerations and management implications during PBSC collection and transplantation. Our report focuses on temperature safeguards throughout the apheresis workflow, expanding the existing literature and highlighting procedural considerations.

## 2 Case Presentation

A 49‐year‐old African American man presented with palpitations and syncope at an outside hospital and was found to have profound anemia (Hgb 4 g/dL), biochemical evidence of hemolysis, and a direct antiglobulin test (DAT) positive for C3 and for IgG, with no antibodies detected on elution testing. His haptoglobin levels were undetectable, lactate dehydrogenase (LDH) and bilirubin levels were elevated, and testing for COVID and Rhinovirus was positive. Hematology laboratory testing demonstrated cold agglutinin‐related interference affecting complete blood count parameters. Serology identified a cold auto‐anti‐I antibody with a cold agglutinin titer of 512. His clinical picture was thought to be consistent with CA and a component of warm autoimmune hemolytic anemia (WAIHA). He responded transiently to prednisone and rituximab, achieving transfusion independence, but later developed recurrent anemia and persisting transfusion needs.

At our institution, several months later, bone marrow evaluation for his anemia revealed up to 55% plasma cell involvement, a dominant IgA‐kappa monoclonal protein (2.72 g/dL), markedly elevated IgA (5497 mg/dL), and a kappa: lambda ratio of 57.25. Flow cytometry demonstrated a rare, atypical B‐cell population with unusual immunoglobulin expression and negative kappa light chain expression. It was unclear whether this population represented an unrelated CAD‐associated B‐cell clone or was a spurious finding. Serum electrophoresis and immunofixation demonstrated the IgA‐kappa monoclonal protein and a small IgG‐kappa monoclonal protein thought to be the rituximab. Cytogenetics and myeloma FISH demonstrated 1q duplication, hyperdiploidy, *t*(4;14), and IgH rearrangement. Positron emission tomography/computed tomography showed diffuse marrow uptake without focal osseous disease. DAT was positive for C3d and negative for IgG; LDH remained elevated with elevated bilirubin. These findings supported a diagnosis of IgA‐kappa multiple myeloma in a patient with active cold agglutinins.

The patient received four cycles of daratumumab, lenalidomide, bortezomib, and dexamethasone with improvement in hemoglobin (10.6 g/dL) and reduction of IgA levels. Prior to PBSC mobilization, he developed recurrent anemia and elevation in IgA after holding therapy. While there was no difficulty with macroscopic agglutination or difficulty with peripheral blood draws, there was red blood cell agglutination visible on peripheral blood smear. Cold agglutinin titers were rechecked and found to be positive at 64, 4 weeks prior to the apheresis collection. Thermal amplitude studies performed 3 weeks before collection demonstrated persistent reactivity across a broad temperature range, with saline and 22% albumin titers of 256 and 256, respectively, at room temperature; 4 and 128, respectively, at 30°C; and 0 and 8, respectively, at 37°C. The persistence of reactivity at 37°C in 22% albumin indicated residual antibody activity near physiologic temperature.

A multidisciplinary workflow was established to mitigate cold‐induced agglutination across collection, transport, and processing. To minimize risks during mobilization, he received cytoreductive cyclophosphamide followed by G‐CSF and three doses of sutimlimab to optimize hemoglobin and reduce complement‐mediated hemolysis. A cold agglutinin titer was not repeated on the day of collection. PBSC collection was performed in a temperature‐controlled environment, with the apheresis machine and collection system maintained in a warm room (approximately 30°C) prior to each session. The collection was performed at elevated ambient room temperature (30°C), and additional pragmatic measures were employed for thermal control, including the use of warming blankets around the apheresis device to reduce heat loss, with particular attention to the base and to minimizing exposure at access points around the door interfaces. In addition to using a warmer for the return line—set at 41°C, the inlet and return lines were positioned under patient warming blankets, where feasible, to limit ambient cooling. Using a continuous flow centrifugation‐based platform the Spectra Optia version 12 (Terumo BCT, Lakewood, Colorado, USA), with the continuous mononuclear cell collection software, a total of 5.61 × 10^6^ CD34^+^ cells/kg was collected over 2 days (18 and 12 L of whole blood processed on Days 1 and 2, respectively, with respective product volumes of 300 and 197 mL) without agglutination or circuit interruption. Vascular access was maintained using bilateral arm peripheral catheters via a 17 G inlet and an 18 G return line needles. The anticoagulant was citrate with a ratio of 12, and the patient received 2 g of intravenous Calcium Gluconate during each collection procedure. The patient's body temperature ranged from 37.1°C to 37.8°C throughout the two apheresis collections. The two procedures were well tolerated without any complications. The product was transported in a warm container and was kept between warm packs at 37°C at all times during transport and at the Cell Therapy Laboratory. The product was processed as soon as it was received into the laboratory, approximately 10 min from removal from completion of collection to receipt in the processing laboratory. It was washed and centrifuged twice with a 50:50 mixture of human serum albumin (HSA) and Plasma‐Lyte. All processing solutions and centrifuge buckets were warmed to 37°C prior to processing. The centrifuge was prewarmed by lining with eight warm packs at 37°C, for 2 h prior to the initial centrifugation step. All gel packs were warmed in a water bath set to 37°C before use. They were dried and sanitized with isopropyl alcohol just prior to use. The plasma press was prewarmed to 37°C, while the biosafety cabinet and cryomedia syringes were kept at ambient temperature (23°C). The cryopreservation freeze cycle was not modified and began at 22°C. No visible aggregates were observed following product processing, after centrifugation, or before cryopreservation. The collection efficiency was calculated using the CE2 method. The patient's overall CE2 was 29%, which was lower than the typical collection efficiencies observed at our institution (> 40%).

The patient subsequently received high‐dose melphalan (200 mg/m^2^) and, following an extended thawing of the product in a 37°C water bath, he underwent an autologous HPC infusion of 2.03 × 10^6^ CD 34+ cells/Kg. No visible aggregates were observed during postthaw inspection, and there was no difficulty during thawing or infusion. His peri‐transplant course was uncomplicated. At 3 months posttransplant, laboratory testing showed his hemoglobin had increased to 11.2 g/dL without evidence of recurrent hemolysis and with no detectable paraprotein. There was no evidence of active myeloma on the PET/MRI scan. Bone marrow biopsy demonstrated trilineage hematopoiesis and detectable minimal residual disease (MRD) by ClonoSeq (1 cell per million), below the test's FDA‐approved threshold of 10^−5^ for MRD negativity. The previous atypical B‐cell population was not detected. He subsequently began daratumumab and lenalidomide maintenance therapy.

### 2.1 Outcome

**FIGURE 1:**
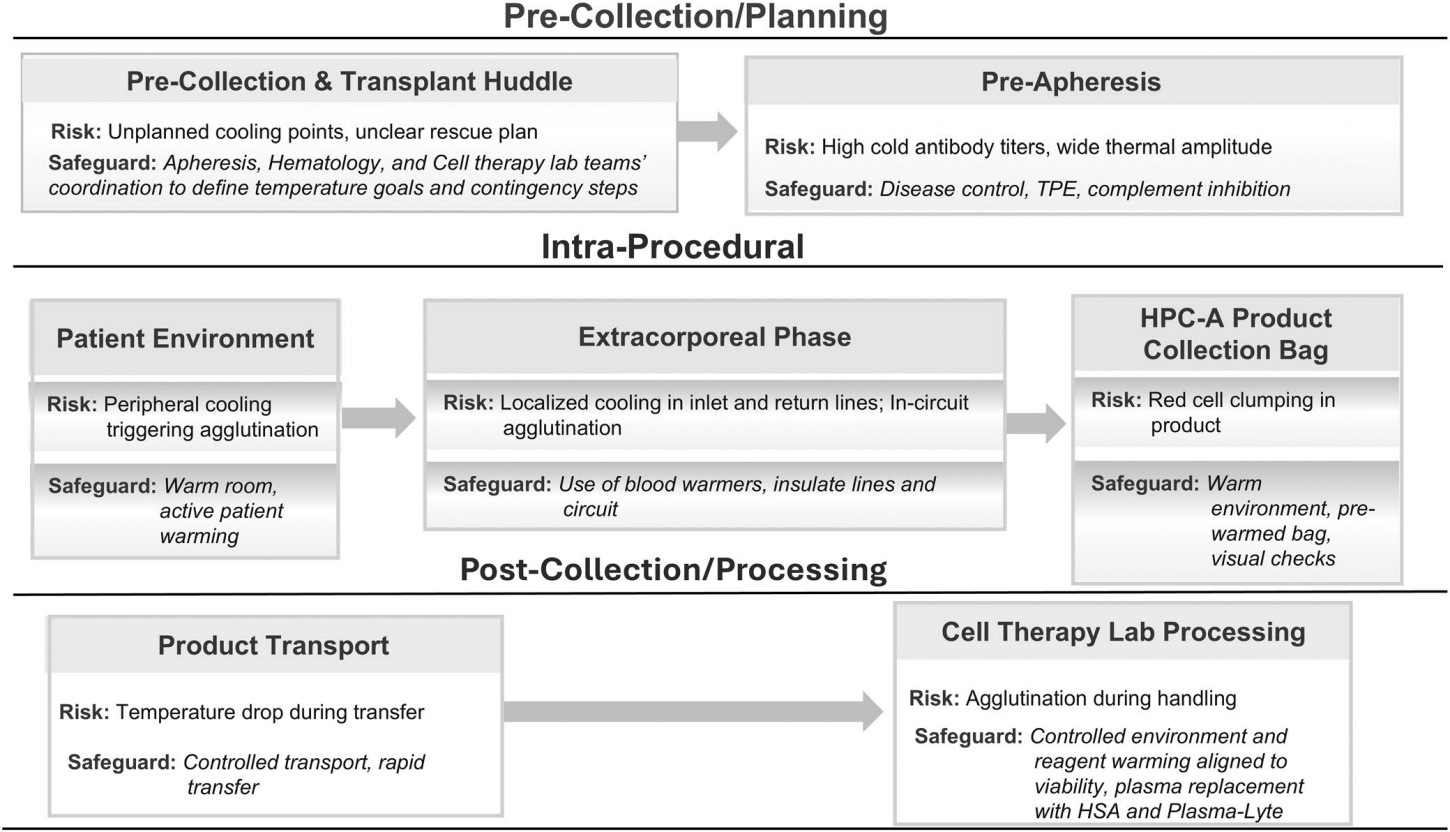
Temperature safeguards across the peripheral blood stem cell collection workflow in cold agglutinin disease. Cold‐reactive antibodies pose a risk of agglutination at multiple stages of peripheral blood stem cell collection. This schematic illustrates key temperature‐related vulnerabilities and corresponding safeguards applied in the index case and in the published literature, across the workflow, from patient preparation through extracorporeal circulation, collection, transport, and cell therapy processing. Emphasis is placed on proactive environmental warming, circuit temperature control, continuous visual monitoring, and multidisciplinary coordination to mitigate cold‐induced red blood cell agglutination. HPC‐A: hematopoietic progenitor cell apheresis; HSA: human serum albumin; TPE: therapeutic plasma exchange.

Following transplantation, the patient became transfusion‐independent, and cold‐induced symptoms resolved. As at 14 months posttransplant, he remains in remission from both CAD and multiple myeloma at follow‐up. The findings from the index case and from previous reports on PBSC in CA settings are shown in Table 1. Our multidisciplinary temperature control strategies across the HPC‐A workflow, integrated with previously published strategies, are shown in Figure 1.

**TABLE 1:** Summary of index case and published cases of PBSC collection in cold agglutinins.

Case and year	Population and diagnosis	Type of transplant	CA Titers	Mobilization regimen	Precollection CAD mitigation	Collection parameter (device; collection sessions)	Apheresis collection safeguards	HPC‐A Product safeguards	CD34 yield × 10^6^/kg	Transplant outcome
Index case	49 M; IgA myeloma	Autologous	512 (30°C); *Repeated before collection:* 128 (30°C) 8 (37°C)	G‐CSF	Cytoreductive cyclophosphamide, sutimlimab	Spectra Optia; 2	Warm room, warming blankets on patient and on inlet and return line, insulation of apheresis device with warming blankets, use of warmer on return line	Environmental warming, warm transport container, prewarmed centrifuge and plasma press (37°C); washing with 50:50 HSA and Plasma‐Lyte warmed to 37°C	5.61	Successful engraftment and remission
Crowther 2006	62 M; DLBCL	Autologous	512 (20°C) 4 (37°C)	G‐CSF	TPE × 1	Cobe Spectra; 1	Warm room, warming blanket, overhead heater above apheresis machine	Collected in 37° water bath, transported in 37° water bath, prewarmed laboratory equipment and reagents	6.3	Successful engraftment and remission
Badami 2017	61 F; Marginal‐zone NHL	Autologous	64 (4°C) 16 (16°C) 2 (30°C)	G‐CSF	TPE × 2	NR; 2 sessions	Warming blanket, hat, warmers on return/inlet lines, prewarmed fluids	37° water bath prior to and during processing	3.4	Successful engraftment, primary disease relapse
Morigi 2021	59 F; aggressive NHL	Autologous	16 (NR°C) pre‐TPE	G‐CSF and Plerixafor	TPE × 1	COM.TEC; 1	Warm room	Transported and processed in warm room	7.96	Successful engraftment and remission
Castonguay 2025	61 M; DLBCL	Autologous	> 4096 (4°C) 128 (37°C)	G‐CSF	TPE × 5 + Eculizumab	NR; 1	Warmed equipment and fluids: warm room, warming blanket, warmers on return/inlet, apheresis device; similar conditions for graft manipulation	Transported in warm box and processed between two bags of warmed beads in warm room, prewarmed centrifuge and calibration bags, warmed Plasma‐Lyte wash, preheated plasma cryopreservation, same thermal conditions for infusion as collection	6.73	Ongoing hemolysis requiring multiple transfusions during aplasia. Back to baseline following marrow recovery with transfusion independence except during viral infections
Yuan 2020	67 M; DLBCL (retrospective CAD diagnosis)	Autologous	256 (4°C) 8 (22°C) 1 (30°C)	None	None	NR; 1	None	None	NR	NR
Thompson 2023	66 M; PV/MF (CAD in recipient)	Allogeneic	> 512 (NR°C)	NR	Not applicable	NR; NR	Not applicable	Extended product warming procedure prior to infusion	NR	Successful engraftment
	62 M; Plasmacytoma and amyloidosis	Autologous	> 512 (NR°C)	NR	TPE × 1	NR; 2	Warm room, warmers on return/inlet	Prewarmed human albumin resuspension, extended warming procedure prior to infusion	NR	Successful engraftment
	65 M; MF (Concern for CA in donor)	Allogenic	NR	NR	NR	NR; NR	NR	Extended warming prior to infusion	NA	Successful engraftment
Pawson 2025	58 F; low‐grade LPD	Autologous	32 (4°C) 2 (22°C)	G‐CSF	TPE × 1	NR; 1	Warmers on return/inlet, heat pads on patient arms	Kept at room temperature as longs as possible prior to cryopreservation, Extended warming prior to infusion	10.96	Did not undergo transplant
	40 F; low‐grade LPD	Autologous	64 (4°C)	G‐CSF	TPE × 1	NR; 1	Warmers on return/inlet, heat pads on patient arms	Kept at room temperature as long as possible prior to cryopreservation, Extended warming prior to infusion	9.2	Successful engraftment

Abbreviations: CA, cold agglutinin; CAD, cold agglutinin disease; DLBCL, diffuse large B‐cell lymphoma; G‐CSF, granulocyte colony stimulating factor; HPC‐A, hematopoietic progenitor cell apheresis; HSA, human serum albumin; LPD‐lymphoproliferative disorder; MF‐ myelofibrosis; NHL, non‐Hodgkin lymphoma; NMDP, National Marrow Donor Program; NR‐not reported; PBSC, peripheral blood stem cell; PV, polycythemia vera; TPE, therapeutic plasma exchange.

## 3 Discussion

CAD is most commonly mediated by IgM autoantibodies, which bind to RBCs at low temperatures, activating the classical complement pathway and resulting in hemolysis, with a well‐described association with clonal B‐cell lymphoproliferative disorders. IgA‐mediated CAD, in contrast, is rarely described in medical literature. One report describes a patient with IgA multiple myeloma presenting as a cold‐induced hemolytic transfusion reaction [9]. In the index case, although it is tempting to unequivocally attribute the cold agglutinin to IgA myeloma, IgA has not been demonstrated to activate complement through the classical pathway, unlike IgM. IgM‐k, usually implicated, enables the potent activation of the classical complement pathway that defines the disease in CAD due to its pentameric structure, which enables efficient agglutination at low temperatures, a feature lacking in IgA. In diagnostic testing, the DAT is positive with anti‐C3d, reflecting complement deposition on RBCs. In the broader context of autoimmune hemolytic anemia (AIHA), diagnostic pitfalls may occur with IgA autoantibodies, but these descriptions have been documented in WAIHA, not specifically in CAD [10, 11]. Previous research on pure IgA‐associated AIHA found that IgA‐coated RBCs did not activate complement but induced phagocytosis [12, 13]. However, because this phagocytosis was only moderate compared to that caused by IgG‐coated RBCs, it is unclear whether the human IgA Fc receptor‐ FcαRI‐mediated erythrophagocytosis is the main pathogenic mechanism in IgA‐induced AIHA. Another documentation of a severe AIHA due to IgA found that the splenic trapping and sequestration of agglutinated RBCs was the etiopathogenic mechanism in one patient [14]. Sefland et al., in their report on a patient with IgA multiple myeloma and CAD, propose that the IgA class does not cause CAD and that non‐IgM‐associated CAD should prompt a search for the coexistence of two unrelated B‐cell clones [15]. The index patient had a history of COVID infection at the time of initial diagnosis with laboratory findings of active CA and a component of WAIHA, suggestive of a CAS and a WAIHA. However, a subsequent diagnosis of IgA‐kappa myeloma and a finding of an atypical B‐cell population of undetermined significance, with active CA, is suggestive of a CAD, making the diagnosis even more complex. Nonetheless, prior to his autologous transplant, the patient's CA responded to sutimlimab, a complement inhibitor, suggesting a complement‐mediating effect of his cold autoantibodies.

Thermal strategies are central to safe PBSC collection in the presence of CA. The clustering of risks across multiple procedural phases warrants the need for continuous temperature control throughout the workflow (Figure 1). Warming of the extracorporeal circuit, minimizing cold exposure during apheresis, temperature‐controlled transport, and coordinated thawing protocols reflect best optimization practices described by Gillies et al. [5], Castonguay et al. [4], and others [1–3, 16]. Our approach builds upon these observations by integrating mitigation strategies within a structured, workflow‐based framework spanning the various phases of collection and product processing. Procedural warming reduces in‐line agglutination, prevents hemolysis, apheresis circuit alarms, and flow obstruction, and allows uninterrupted collection. In our case, these thermal optimization measures were implemented proactively, and no cold‐related complications occurred. Castonguay et al. detailed autologous transplantation in severe CAD with temperature optimization and preprocedural plasma exchange. Their patient had also received Eculizumab, a complement inhibitor [4]. We considered the use of therapeutic plasma exchange for the reduction of the CA; however, we opted to delay PBSC collection and treat the patient's disease with chemotherapy and sutimlimab, a complement inhibitor that binds to complement protein component 1, s subcomponent, blocking the classical complement pathway. While sutimlimab is increasingly used in the management of CAD, there are limited published reports describing its integration into peri‐procedural strategies for PBSC collection and processing.

Product‐level vigilance is supported by prior documentation of RBC clumping within HPC‐A collection bags, highlighting that agglutination may occur even in the absence of overt circuit instability [6]. In addition, recent literature evaluating warming strategies for HPC‐A products at risk for cold agglutination demonstrates that controlled warming can preserve cell viability without compromising product quality [7, 8]. Despite the reduced collection efficiency noted in the index report, a cumulative CD34+ cell dose that exceeded the minimum target for autologous transplantation was successfully collected. Given the single‐patient nature of this report, we cannot determine whether the lower CE2 was attributable to CAD, procedural modifications implemented to maintain normothermia, or other patient‐specific factors.

Successful autologous transplantation is feasible in CAD. Although reports are limited, previous cases support the concept that PBSC transplantation can be safely performed when appropriate precautions are taken [1, 2, 3, 4, 16]. Our patient achieved prompt neutrophil and platelet engraftment, without evidence of delayed graft function or hemolytic complications. The clinical course reinforces that CA alone should not preclude autologous transplant when other disease‐related factors support its use.

This case expands the existing literature by describing CA in a patient with IgA‐kappa myeloma requiring autologous PBSC transplantation, a circumstance not widely reported. It highlights that the disease in our patient is not a single entity but a spectrum of antibody‐mediated disorders in which the immunoglobulins, degree of complement activation, and associated hematologic disease influence both risk and management. The PBSC mobilization and HPC‐A collection required careful planning and multidisciplinary coordination of care due to the persistent risk of cold‐induced agglutination throughout the apheresis and cell‐processing workflow. The mobilization process also included the use of sutimlimab to improve hemoglobin levels prior to apheresis, which has not been reported previously. A limitation of our approach is that the warming measures employed were intended to mitigate, and not completely eliminate the risk of cold agglutinin‐mediated agglutination, because the last thermal amplitude testing demonstrated persistent reactivity at 37°C. Although the reactivity at 37°C was substantially weaker than at lower temperatures (22% albumin titer 8 at 37°C versus 128 at 30°C and 256 at room temperature) before treatment with sutimlimab, residual antibody activity at near‐physiologic temperatures may have remained; however, this was not evident clinically. As cellular therapies continue to be increasingly used, recognition of atypical serologic patterns and early planning and implementation of thermal strategies will be essential to avoid procedural complications. Close collaboration between hematology, transfusion medicine, and apheresis teams is critical to ensure safe collections and successful transplantations and engraftments.

## 4 Conclusion

Autologous stem cell transplantation can be safely performed in patients with CA, including those with atypical immunoglobulin patterns such as IgA‐kappa myeloma, when proactive thermal precautions are employed. This case contributes to the limited literature and provides practical guidance for centers encountering cold‐sensitive hemolysis during PBSC mobilization, collection, processing, and infusion.

## Funding

The authors have nothing to report.

## Ethics Statement

The authors have nothing to report.

## Consent

Informed consent for publication of de‐identified clinical information was obtained from the patient.

## Conflicts of Interest

The authors declare no conflicts of interest.

## Data Availability

The data that support the findings of this study are available on request from the corresponding author. The data are not publicly available due to privacy or ethical restrictions.
